# Influences of Kinesio Taping with Therapeutic Exercise in Patients with Low Back Pain

**DOI:** 10.3390/healthcare9080927

**Published:** 2021-07-22

**Authors:** Kyoung-sim Jung, Jin-hwa Jung, Tae-sung In, Hwi-young Cho

**Affiliations:** 1 Department of Physical Therapy, Gimcheon University, Gimcheon 39528, Korea; 20190022@gimcheon.ac.kr; 2 Department of Occupational Therapy, Semyung University, Jecheon 27136, Korea; otsalt@semyung.ac.kr; 3 Department of Physical Therapy, College of Health Science, Gachon University, Incheon 21936, Korea

**Keywords:** taping, low back pain, endurance

## Abstract

The aim of this study was to evaluate the effect of core stability exercise combined with Kinesio taping on pain, endurance, and balance in patients with lower back pain (LBP). 46 patients with LBP were recruited and randomly allocated into the core stability exercise with taping (CSET) group and the core stability exercise (CSE) group. All participants performed core stability exercises for 40 min/day, 5 times/week for 8 weeks, and additional Kinesio taping was applied to the lower backs in the CSET group. The primary outcome measure was the pain intensity using the visual analog scale, and secondary outcome measures were trunk endurance and balance using the Biering-Sorensen test and force plate, respectively. After the intervention, the CSET group showed significant improvements in pain and postural balance compared to the CSE group (*p* < 0.05). However, there was no significant difference in trunk endurance between two groups (*p* > 0.05). This study found that core stability exercise was effective in reducing pain and enhancing balance in patients with LBP, and demonstrated that the application of additional Kinesio taping further increased these effects. Therefore, we recommend that core exercise combined with Kinesio taping may be used to improve the pain and postural balance of patients with LBP in clinics.

## 1. Introduction

Deep-trunk musculature including the multifidus, transverse abdominis (trA), and internal oblique muscles are the primary stabilizers of the lumbar spine and trunk during movement and are known as postural control muscles [1]. In patients with low back pain (LBP), the pattern and amount of activation in deep-trunk musculature are not properly induced, and spinal stability is decreased [2]. These changes also cause uncontrolled movement in the lumbar spine in LBP patients, resulting in a vicious cycle of worsening pain and dysfunction [3]. In addition, studies have reported that patients with LBP have higher pain intensity than those without pain when sitting for a long time [4], and the back muscles tend to tire out easily [5]. Therefore, a training method that promotes deep trunk muscle activation could reduce pain and stabilize the lumbar spine in patients with LBP.

Core stability exercise aims to restore and strengthen trunk stability and functional movement by retraining the co-activation of the muscles in the abdomen, back, pelvis, and chest [6] Therefore, it is believed that this exercise could correct the movement control pattern and activity amount of the deep-trunk musculature in patients with LBP, and through this, proper spinal movement control and stability could be restored when performing functional movements [7]. However, it remains controversial whether it is more effective than the other types of therapeutic exercise in patients with LBP [8].

The taping method has been used to manage and treat symptoms of musculoskeletal injuries without side effects in hospitals, and it has recently been used as a treatment option to manage and prevent symptoms caused by LBP, shoulder joint injury, and various sports injuries [9]. Although there are various taping application methods, Kase’s method using elastic taping is the most commonly used, and this method applies taping to the skin of the relevant muscle while the target muscle is stretched as much as possible [10]. Taping stimulates mechanoreceptors in the skin to activate the gate control system and thereby relieve pain. In addition, various therapeutic effects such as an increase in joint ROM and muscle strength, decrease in muscle fatigue, decrease in edema, and increase in exercise capacity have been confirmed [11]. In addition, Murray et al. [12] suggested that taping could enhance proprioception by increasing stimulation of the skin’s mechanoreceptors. A study has reported that taping could improve muscle endurance in patients with LBP [13]. However, Chang et al. [14] suggested that a single application of taping had no clinically significant effect on improving muscular endurance in patients with LBP, and it was necessary to confirm the effect of repeated application of therapeutic exercise and additional taping on pain and muscle endurance. Furthermore, a review study suggested that taping alone had no significant effect on reducing pain and increasing functional movement in patients with LBP, whereas the application of taping in addition to other therapeutic interventions could further increase the existing effect [15]. Although many previous studies have reported the effect of taping on various symptoms following LBP, most have identified the effect of a single application. Studies on the effect of repeated application of taping on LBP are lacking, and the effect of repeated application of therapeutic exercise with taping is still unclear. In addition, most of the studies related to interventions for LBP focus on pain and motor dysfunction among various symptoms.

Therefore, this study aimed to suggest an effective therapeutic intervention method for LBP patients by investigating the effects of core stability exercise combined with Kinesio taping on pain, endurance, and balance.

## 2. Materials and Methods

### 2.1. Participants

Forty-six patients with LBP were recruited from J Hospital in South Korea. All participants included in the study experienced LBP for ≥3 months, were aged between 18 and 65 years, had a visual analog scale (VAS) score of ≥3, could maintain their standing posture independently for ≥30 s, and could understand and follow the researcher’s instructions [16]. The following patients were excluded: patients with radiculopathy, those with history of lumbar fracture or surgery, those taking medications affecting posture control, gait, or pain, those with lumbar-related diseases, those who had undergone any physical therapy 8 weeks before recruitment, and those taking corticosteroids 2 weeks before recruitment [15]. We used G*power 3.1.9.4 software (Heinrich-Heine-University Düsseldorf, version 3.1.9.4, Düsseldorf, Germany) to calculate the sample size. In the present study, the mean power was set at 0.8 and the alpha error at 0.05. The effect size was set to 0.8326109 based on the pilot study (12 subjects). The analysis of G*power software shows that at least 19 participants would make an acceptable group sample size for each group; thus 46 participants were recruited in consideration of drop-out.

### 2.2. Protocol

In this study, 46 patients fulfilling the inclusion criteria were selected and randomly assigned into the core stability exercise with taping (CSET) group and the core stability exercise (CSE) group. To perform random allocation, each subject, without information about the study, picked a number from a sealed envelope.

Both groups performed core stability exercises for 40 min a day, 5 times a week for 8 weeks. This study used a single-blinded method to increase the reliability of the evaluation. Training and measurements were performed by different physical therapists with over 5 years of clinical experience. Informed consent was voluntarily obtained from all patients before participation in this study, which was approved by the Institutional Review Board (IRB) of Gachon University (IRB no. 1044396-202007-HR-140-01).

### 2.3. Intervention

Core stability exercises were performed in the order of independent isometric contraction of the trA and multifidus, co-contraction and functional tasks of deep body commuters, functional tasks with loads, and functional tasks with unstable surfaces. At the beginning of training, starting with isometric contraction of the core muscles with low intensity, the intensity was gradually increased until the functional task was performed, and four phases were performed for 10 sessions each. In Phase 1, independent isometric contractions of trA and multifidus were performed, and in Phase 2, co-contraction of deep trunk muscles was performed. In Phase 3, the exercise of Phase 2 was performed with external load applied to the wrist and ankle, and in Phase 4 functional tasks on unstable surfaces were included. Five minutes each of warm-up and cool-down were performed before and after the exercises, respectively. All functional movements were performed in the neutral position of the lumbar spine [8] (Supplementary Figure S1).

**Figure 1:**
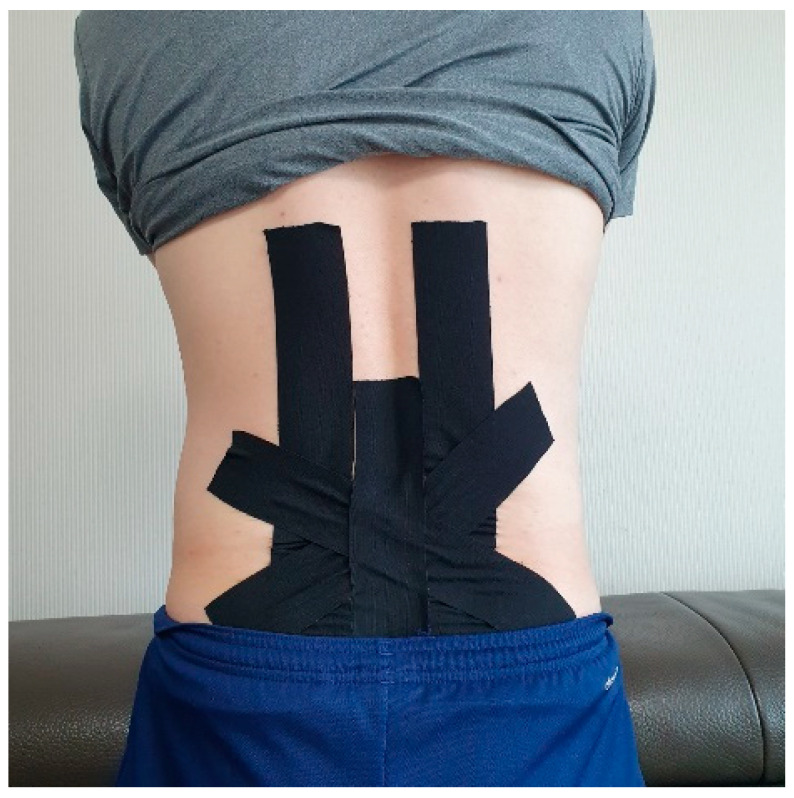
Taping application in CSET group.

Taping was applied additionally in the CSET group. For taping, an elastic Kinesio tape with a 5 cm width was used, and the tape was changed once every 3 days. Two I-shaped strips were cut by measuring the length from PSIS to T12 in trunk flexion. In the upright sitting position, the bases of the strips were placed on both sides of the PSIS, and the strips were attached without tension on both erector spinae muscles, with the trunk flexed as much as possible. Another 3 I-shaped strips were attached to the lower lumbar in the form of an asterisk. The central part where the strips overlap was attached without tension, and the rest part was attached by applying the maximum tension [17] (Figure 1).

### 2.4. Outcome Measurements

Pain, endurance, and balance were measured before the intervention and 1 day after the last intervention. The VAS was used to measure pain intensity. The score ranges from 0 to 100, and it was measured in units of length up to the location where the patient indicated the level of pain. VAS is known to have a high sensitivity to measure pain and is proportional to the pain experienced by the patient. This is the most widely used measurement method, exhibiting good reliability for chronic as well as acute pain [18].

The Biering-Sorensen test was used to measure the endurance of the trunk musculature. This method showed high test-retest reliability with an intra-class correlation coefficient of 0.88 in the evaluation of the isometric trunk muscle endurance [19]. For the examination, the participant was made to lie down with the anterior superior iliac spine on the upper edge of the examination table. The pelvis, knees, and ankles were fixed to the examination table with three straps. The participant was instructed to cross arms in front of the chest and maintain the upper body in a horizontal position isometrically for as long as possible. Prior to the test, the participant was made to lean against another table of the same height, and the table was removed at the start of the test.

A force plate (PDM Multifunction Force Measuring Plate; Zebris, Germany, 2015) was used to measure the postural sway. Participants were asked to stand in a comfortable position with their arms at the side and stare at a point of 15-cm diameter placed 3 m away. The total sway length was measured for 30 s. The average value was obtained after each measurement was repeated thrice [20].

### 2.5. Data Analysis

Normality of variables was assessed using the Shapiro-Wilk test. The independent *t*-test and chi-square test were used for continuous variables and categorical variables, respectively, to compare the baseline characteristics of the participants of the CSET and CSE groups. The paired *t*-test was used to compare changes in the baseline values within each group, and the independent *t*-test was used to compare the before and after changes between the groups. The level of statistical significance was set at 0.05. Statistical analyses were performed using Statistical Package for the Social Sciences (SPSS version 21.0).

## 3. Results

### 3.1. General Characteristics

The general and medical characteristics of all participants in the CSET and CSE groups were homogenous (Table 1).

### 3.2. Changes of Pain Intensity

The VAS score significantly decreased in the CSET and CSE groups after the intervention (*p* < 0.05). The improvement in the VAS score of the CSET group (mean change, −30.39 ± 13.27 score) was more significant than that of the CSE group (mean change, −14.26 ± 10.51 score) (Table 2).

### 3.3. Changes of Endurance

Trunk endurance significantly increased in the CSET and CSE groups after intervention (*p* < 0.05); however, there was no significant difference between the groups (Table 3).

### 3.4. Changes of Balance

After training, the CSET and CSE groups showed a significant decrease in postural sway (*p* < 0.05), but the decrease in postural sway in the CSET group was more significant. The changeable amount before and after training was −35.72 ± 15.88 and −24.46 ± 17.35 cm, respectively (Table 4).

## 4. Discussion

We confirmed the effect of core stability exercise and additional Kinesio taping on pain in patients with low back pain. As a result of the present study, both groups showed significant pain improvement after intervention, and in particular the application of Kinesio taping further increased the effect of therapeutic exercise (Table 2). According to previous studies, taping could promote blood flow, and activates the pain suppression system by stimulating afferent nerve fibers in the soft tissue of the applied area [21,22]. We presumed that the pain relief of the subjects may have been due to this effect of taping. Interestingly, Ostelo et al. suggested that the level of minimal clinically important difference in VAS was 15 points in patients with LBP [23]. In our VAS results, after intervention, the CSET group showed a change of 30.39 ± 13.27 points, while the CSE group showed a change of 14.26 ± 10.51. As such, we confirmed that the CSE group did not reach a minimal clinically important difference, but the CSET group did. Through this, it is suggested that additional taping application is more effective than applying only core stability exercise to manage pain in patients with LBP.

LBP Patients may have decreased levels of muscular endurance in the trunk region due to various causes, such as increased metabolic levels due to prolonged muscle contraction or spasms [24], decreased muscle coordination [25], atrophy, and inhibition of the paraspinal muscles due to pain [25,26]. Core stability exercises are known to improve the coordination of agonists and antagonists through muscle training [6]. Furthermore, Shih et al. reported that taping increased muscle activity [27]. Therefore, in this study, the change in endurance of the trunk muscles was measured after training. However, there was no significant difference between the groups. These results indicate that the training method is not sufficiently effective in improving the endurance, or that the measurement method does not accurately reflect the improvement. The Biering-Sorensen test measures the time required to maintain the upper body without support in the prone position, and it is a widely used method to evaluate the endurance of trunk muscles [5]. However, Jubany et al. suggested that the dysfunction in patients with LBP could be hidden or weakened because the muscles are evaluated in a posture that is quite different from that of daily living activities [28]. Moreover, most of the studies examining the effect of taping on muscle activity confirmed the immediate effect with the tape attached, but there is insufficient evidence regarding the long-term treatment effects on muscle activity or muscle endurance. Chang et al. reported that endurance increased only when taping was applied, and the posture holding time decreased after its removal [14]. In addition, there was no significant change due to taping in a study investigating the electromyography characteristics of the paravertebral muscles after taping for 7 days in patients with LBP [29].

This study also measured the changes in postural sway after training. The results revealed that the CSET group showed significant improvement as compared to the CSE group. This could be attributed to the improvement in proprioception. Studies have reported that LBP patients have decreased proprioceptive sensation and poor balance ability [20,30]. Taping increases the input of proprioceptive sensation through the tension of the tape and improves perception of body movement even after the tape is removed [31,32]. In a study comparing balance by attaching a tape to the back muscles of patients with LBP, it was found that the left and right weight distribution was symmetrically improved than that of participants without the tape [17]. Akbari et al. suggested that the modification of the proprioceptive mechanism caused by taping is also effective in reducing the negative effect of fatigue on postural control [33]. Muscle fatigue due to postural imbalance causes the body to move away from the center of gravity, and thus postural sway increases by using a compensatory posture strategy to maintain an upright posture [34].

This study investigated the effects of core stability exercise combined with taping on pain, endurance, and postural sway in patients with LBP. The results revealed a significant improvement in pain and postural sway in both two groups. However, these results cannot be generalized because the sample size was small in this study, and the actual proprioception was not measured. Therefore, future studies are necessary to confirm the effects of taping combined with various treatments on the proprioception of the trunk and the activity and cross-sectional area of the trunk musculature.

## 5. Conclusion

Our results support the study reporting that CSE is effective for pain management in patients with LBP, and also prove that the additional application of taping to therapeutic exercise can further improve pain and balance ability in patients with LBP. Through this, we propose that taping could be used in combination with therapeutic exercise to improve the pain and balance ability of patients with LBP in clinics and sports centers.

## Figures and Tables

**Table 1:** General characteristics of the subjects (*n* = 46).

Variables	CSET Group (*n* = 23)	CSE Group (*n* = 23)	*p*
Sex (Male/Female)	14/9	13/10	0.765 ^b^
Age (years)	48.17 ± 0.57 ^a^	48.09 ± 0.60	0.618 ^c^
Height (cm)	169.26 ± 6.48	168.78 ± 8.12	0.826 ^c^
Weight (kg)	62.78 ± 11.11	61.74 ± 9.21	0.730 ^c^
Duration of LBP (months)	9.26 ± 3.60	10.61 ± 4.12	0.730 ^c^

Note. ^a^ mean ± standard deviation, ^b^ chi-square test, ^c^ independent *t*-test. LBP; low back pain.

**Table 2:** Subject scores of pain intensity before and after intervention.

	CSET Group			CSE Group			
	Pre	Post	Difference	Pre	Post	Difference	*p*
VAS (mm)	55.43 ± 15.67	25.04 ± 8.70	−30.39 ± 13.27 *	53.57 ± 11.80	39.30 ± 11.51	−14.26 ± 10.51 *	0.000

Note. * Significant differences between pre and posttest (*p* < 0.05).

**Table 3:** Subject scores of trunk endurance before and after intervention.

	CSET Group			CSE Group			
	Pre	Post	Difference	Pre	Post	Difference	*p*
Trunk endurance (s)	84.96 ± 20.19	102.78 ± 19.07	17.83 ± 7.90 *	89.35 ± 27.72	104.17 ± 26.24	14.83 ± 7.09 *	0.182

Note. * Significant differences between pre and posttest (*p* < 0.05).

**Table 4:** Subject scores of balance before and after intervention.

	CSET Group			CSE Group			
	Pre	Post	Difference	Pre	Post	Difference	*p*
Total sway length (cm)	265.77 ± 50.63	230.69 ± 48.62	−35.72 ± 15.88 *	254.23 ± 67.76	229.77 ± 64.52	−24.46 ± 17.35 *	0.036

Note. * Significant differences between pre and posttest (*p* < 0.05).

## Data Availability

The data presented in this study are available on request from the corresponding author. The data are not publicly available due to ethical restrictions.

## Supplementary Materials

The following are available online at https://www.mdpi.com/article/10.3390/healthcare9080927/s1, Figure S1: Protocol for core stability exercise.
