# Supplementary material for: Influences of Kinesio Taping with Therapeutic Exercise in Patients with Low Back Pain
Source: Healthcare (Basel). 2021 Jul 22;9(8):927. doi: 10.3390/healthcare9080927 (PMC8394962; doi:10.3390/healthcare9080927)
Supplement: Supplementary file 1 [file healthcare-09-00927-s001.zip › healthcare-1275013-supplementary.pdf]

Figure S1. Protocol for core stability exercise

<table>
<tr>
<td rowspan="6">Phase 1(10 sessions)
:Independent isometric contraction of the transversus abdominis (trA) and multifidus</td>
<td>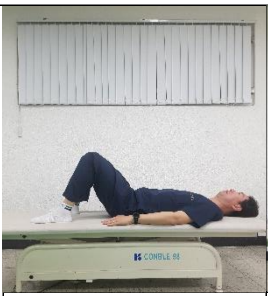</td>
<td>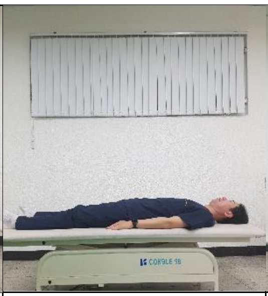</td>
<td>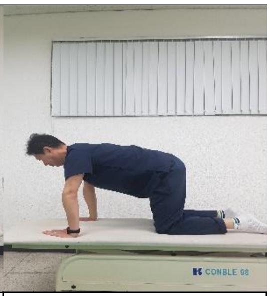</td>
</tr>
<tr>
<td>hooklying</td>
<td>supine</td>
<td>quadruped</td>
</tr>
<tr>
<td colspan="3">Isolated isometric contraction of TrA: Drawing the belly button toward the spine without moving the back</td>
</tr>
<tr>
<td colspan="3">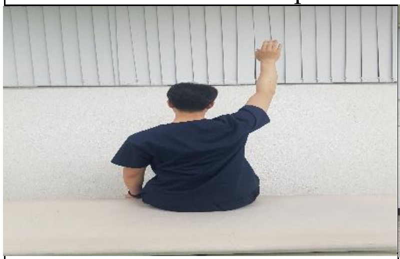 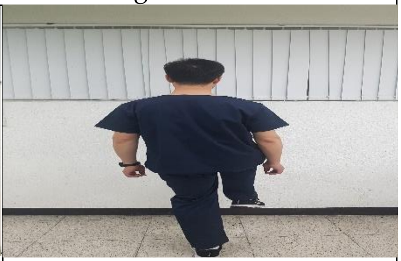</td>
</tr>
<tr>
<td colspan="3">Sitting: Lift the opposite arm while feeling the multifidus muscle contracting
Standing: Stepping your feet alternately while feeling the multifidus muscle contracting</td>
</tr>
<tr>
<td colspan="3">Isolated isometric contraction of Multifidus muscle</td>
</tr>
<tr>
<td rowspan="6">Phase 2 (10 sessions)
:Co-contraction and functional tasks of the deep trunk muscles</td>
<td colspan="3">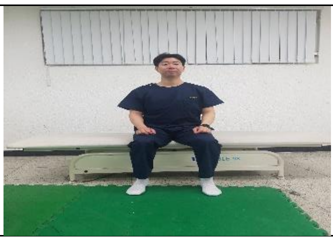</td>
</tr>
<tr>
<td colspan="3">Co-contraction of lumbar stabilizing muscle: sitting</td>
</tr>
<tr>
<td colspan="3">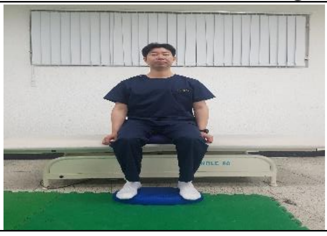</td>
</tr>
<tr>
<td colspan="3">Co-contraction of lumbar stabilizing muscle: maintaining the co- contraction of the lumbar stabilizing muscles while sitting on an unstable surface</td>
</tr>
<tr>
<td colspan="3">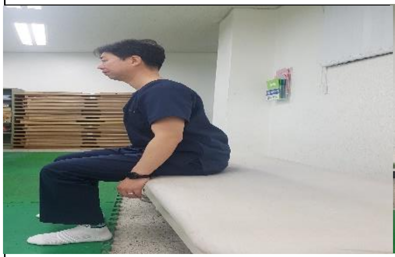 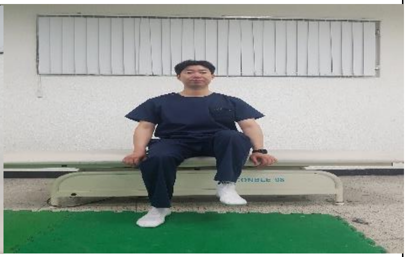</td>
</tr>
<tr>
<td colspan="3">Segmental movement of the thoracic or hip joints: Segmental movement of the thoracic or hip joints while maintaining the co-contraction of the lumbar stabilization muscles</td>
</tr>
</table>

	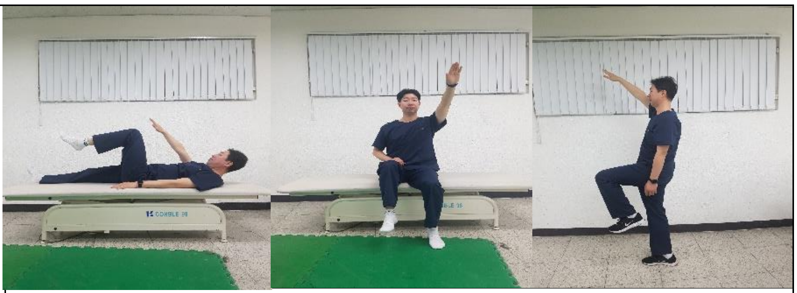<br>Alternately lifting the arms or legs in multiple positions (supine, quadruped, sitting, standing)<br>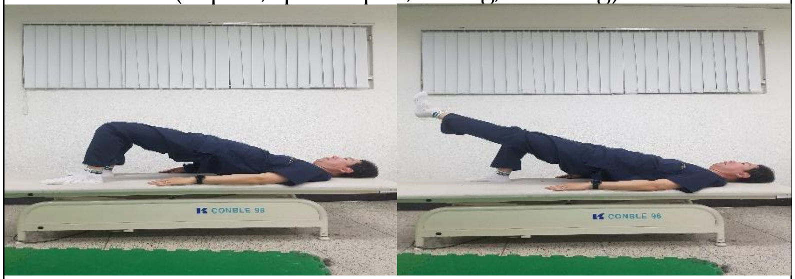<br>Bridging exercise<br>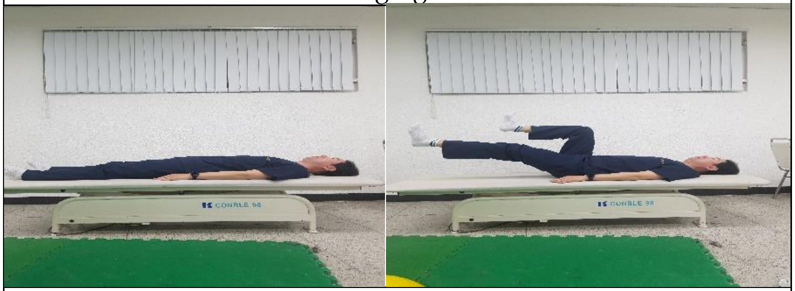<br>Cycling in a supine position
Phase 3 (10 sessions): Functional task with load—the exercises in phase 2 were performed with an external load applied to the wrist and ankle.	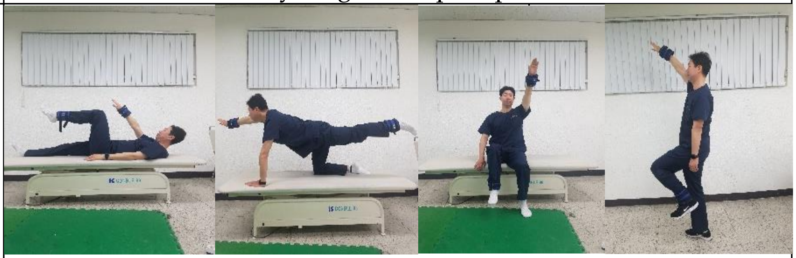<br>Alternately lifting the arms or legs in multiple positions (supine, quadruped, sitting, standing)<br>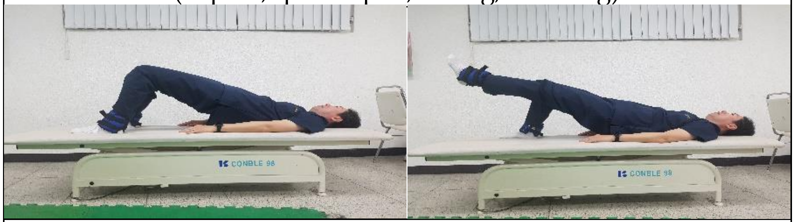<br>Bridging exercise<br>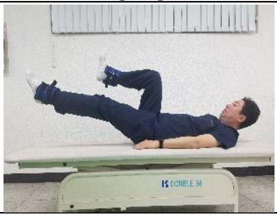<br>Cycling in a supine position

Phase 4 (10 sessions)<br>Functional task with an unstable surface;	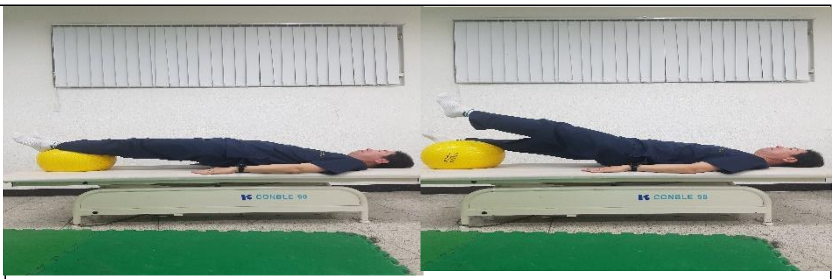<br>Bridging exercise with a balance ball placed under the leg
	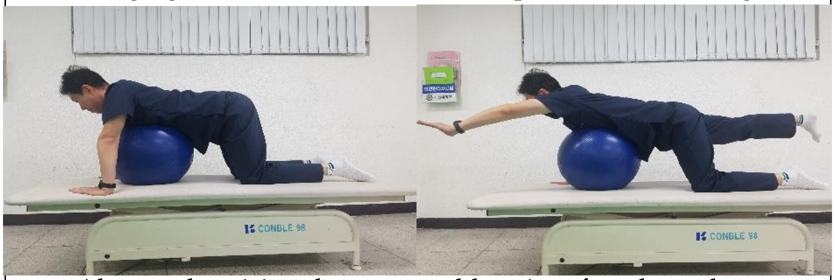<br>Alternately raising the arms and legs in a four-legged posture with the chest rested on the balance ball
	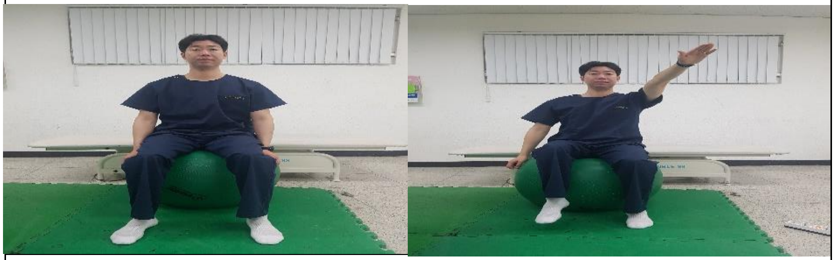<br>Alternately raising the arms and legs while sitting on the balance ball
	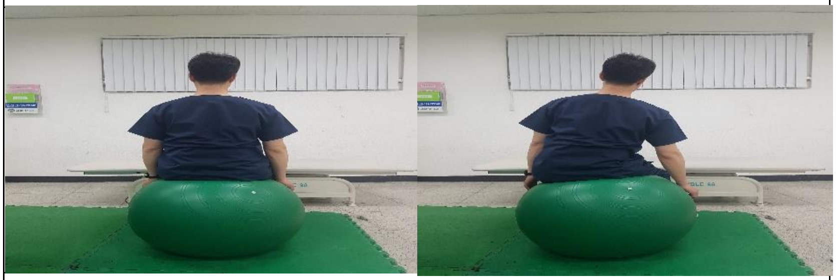<br>Side bending the trunk while sitting on the balance ball.
	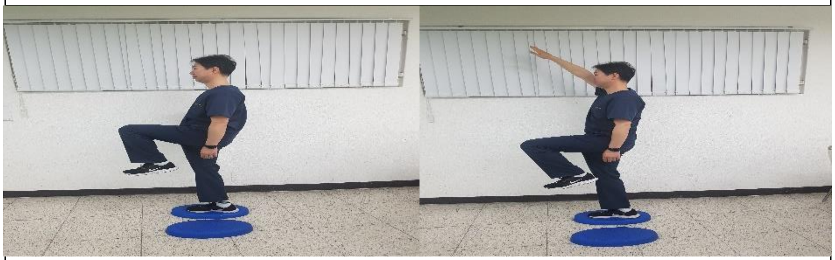<br>Alternately raising the arms and legs while standing on a balance pad
Note. Before and after exercise, 5 minutes of warm-up and cool-down were provided. Subjects were instructed to maintain a neutral lumbar spine position while performing all functional movements.	

*This protocol was modified by adopting the exercise method used in the studies of shamsi et al. [8].
